# Artificial intelligence and radiomics in magnetic resonance imaging of rectal cancer: a review

**DOI:** 10.37349/etat.2023.00142

**Published:** 2023-06-30

**Authors:** Giuseppe Di Costanzo, Raffaele Ascione, Andrea Ponsiglione, Anna Giacoma Tucci, Serena Dell’Aversana, Francesca Iasiello, Enrico Cavaglià

**Affiliations:** University of Campania “L. Vanvitelli”, Italy; ^1^Department of Radiology, Santa Maria delle Grazie Hospital, ASL Napoli 2 Nord, 80078 Pozzuoli, Italy; ^2^Department of Advanced Biomedical Sciences, University of Naples Federico II, 80131 Naples, Italy

**Keywords:** Rectal cancer, magnetic resonance imaging, radiomics, machine learning, artificial intelligence

## Abstract

Rectal cancer (RC) is one of the most common tumours worldwide in both males and females, with significant morbidity and mortality rates, and it accounts for approximately one-third of colorectal cancers (CRCs). Magnetic resonance imaging (MRI) has been demonstrated to be accurate in evaluating the tumour location and stage, mucin content, invasion depth, lymph node (LN) metastasis, extramural vascular invasion (EMVI), and involvement of the mesorectal fascia (MRF). However, these features alone remain insufficient to precisely guide treatment decisions. Therefore, new imaging biomarkers are necessary to define tumour characteristics for staging and restaging patients with RC. During the last decades, RC evaluation via MRI-based radiomics and artificial intelligence (AI) tools has been a research hotspot. The aim of this review was to summarise the achievement of MRI-based radiomics and AI for the evaluation of staging, response to therapy, genotyping, prediction of high-risk factors, and prognosis in the field of RC. Moreover, future challenges and limitations of these tools that need to be solved to favour the transition from academic research to the clinical setting will be discussed.

## Introduction

Colorectal cancer (CRC) represents the third most frequent malignant tumour worldwide in men and women, with rectal cancer (RC) accounting for approximately one-third [1, 2]. Of note, an increasing rate of RC has been demonstrated in patients younger than 50 years [3]. At present, endoscopy is considered the gold standard for RC diagnosis. At the same time, diagnostic imaging plays a pivotal role in evaluating several factors capable of influencing prognosis and therapeutic management, such as local tumour extent (T staging), as well as the presence of lymph nodes (LNs), and presence of metastases (N and M staging) [4].

Endoscopic rectal ultrasound (ERUS) helps diagnose or guide therapy in the early stages of tumours, but it does not add value to locally advanced RC (LARC). Computed tomography (CT) has a fundamental role to assess the presence of metastases, even if it is not capable of a valuable local tumour assessment because of its limited soft tissue resolution [2]. For RC evaluation, magnetic resonance imaging (MRI) is considered the most valuable imaging modality for primary staging and restaging after chemoradiation (CRT) and radiotherapy (RT), guiding any subsequent medical decision [5–8]. In particular, high-resolution MRI (HR-MRI) has been demonstrated to be a game changer in evaluating mucin content, invasion depth, LNs, metastases, extramural vascular invasion (EMVI), and involvement of the mesorectal fascia (MRF) [7, 9–11]. However, conventional HR-MRI has shown some limitations in accurately guiding treatment plans’ development, driving the research towards identifying and validating novel strategies to further increase the value of diagnostic imaging. Artificial intelligence (AI) has been successfully applied in many medical imaging settings, demonstrating that it can automatically recognise complex patterns and provide a quantitative evaluation of medical images [12–16]. In this setting, radiomics has been frequently coupled with AI, and in particular, machine learning (ML) approaches for oncologic imaging, in order to establish models that may improve the accuracy of diagnosis, prognosis, and prediction by extracting and analysing imaging data [17–19]. This review will summarise many critical clinical applications of MRI-based radiomics and AI in the field of RC, including staging, prediction of high-risk factors, genotyping, response to therapy, recurrence, metastases, and prognosis.

## Fundamentals of AI and radiomics

AI is a recently developed branch of computer science that studies and develops systems endowed with the intellectual processes characteristic of human beings [20]. Since it can automatically extract data from diagnostic imaging, make predictions, and mine clinical and radiological information, AI has gained much interest in imaging analysis applied to radiology [16, 18, 21].

ML is a field of AI that can develop mathematical algorithms capable of automatically learning different types of tasks with minimal human intervention [22]. In ML, large datasets previously labeled by scientists are used to train AI algorithms. Accurate performances necessarily need a large amount and significant data variability of the training set. Afterwards, human operators expose algorithms to different unlabeled datasets from multiple sources through a validation process to test and eventually calibrate the algorithm’s output [20].

In unsupervised AI, the algorithm automatically learns from raw data, grouping them into diverse classes according to the characteristics of the training set [23].

ML includes convolutional neural networks (CNNs), neural networks, and deep learning (DL); these last two are the most appreciate tools for creating AI systems for diagnostic imaging [22].

Radiomics is another emerging research in the AI field that involves analysing quantitative data from diagnostic images through automated or semi-automated systems that can be combined with ML techniques to identify new features beyond those obtained by radiologists [24].

Furthermore, the use of radiomics features variation in different imaging techniques throughout the treatment, called “delta radiomics”, has shown promising results in the literature for several oncological purposes [25–29].

## Applications

Radiomics and AI-based systems may improve RC diagnosis, characterisation, prognosis, and treatment, playing a role in tumour segmentation, evaluating histologic aggressiveness risk, or identifying genetic signatures that can aid the diagnosis and prognosis of RC [13, 14]. Furthermore, AI may be a valuable tool to help physicians in developing tailoring RC treatment. AI systems may also create three-dimensional (3D) models that could enhance tumour visualisation during surgery, interventional procedures, or during image-guided treatments, providing a precise lesion evaluation and its relationship with adjacent structures to eventually optimise RT treatments [13, 14, 30, 31].

**Figure 1:**
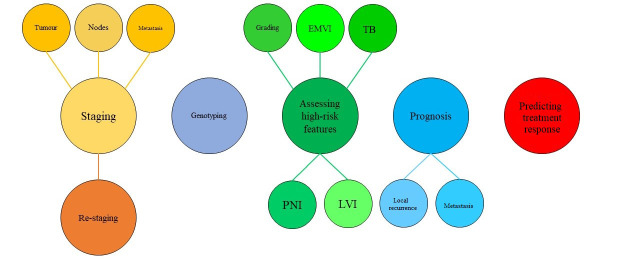
Graphic representation of radiomics and AI main applications in the setting of RC. LVI: lymphatic vascular infiltration; PNI: perineural invasion; TB: tumour budding

In the following sections, recently introduced radiomics and AI approaches explored to increase the value of MRI in managing RC patients will be discussed (Figure 1 and Table 1).

**Table 1:** Main studies on radiomics/AI imaging applications for staging, predicting treatment response, genotyping, and assessing high-risk pathological features and prognosis in the setting of RC management

**References**	**Aim of the study**	**Study design**	**Sample size**	**Main outcome**
[32]	To predict different stages of RC using texture analysis based on diffusion-weighted imaging (DWI) images and apparent diffusion coefficient (ADC) maps.	Retrospective, single center	115	Texture features extracted from DWI images and ADC maps are useful clues for predicting pathological T and N stages in RC.
[33]	To predict tumour pathological features of RC through a T2-weighted image (T2WI) radiomic-based model.	Retrospective, single center	152	T2WI-based radiomics model could serve as pretreatment biomarkers in predicting pathological features of RC.
[34]	To predict the pathological nodal stage of LARC by a radiomic method that uses collective features of multiple LNs in MRI images before and after neoadjuvant CRT (nCRT).	Retrospective, single center	215	Collective features from all rectal LNs perform better than tumour features for the prediction of the nodal stage of LARC.
[35]	To evaluate the predictive performance of radiomics nomogram for the diagnosis of synchronous liver metastases (SLM) in RC patients.	Retrospective, single center	169	The nomogram amalgamating the radiomics signature and clinical risk factors serve as an effective quantitative approach to predict the SLM of primary RC.
[36]	To investigate the value of T2WI radiomic-based MRI in predicting preoperative synchronous distant metastases (SDM) in patients with RC.	Retrospective, single center	177	The proposed clinical-radiomics combined model could be utilized as a noninvasive biomarker for identifying patients at high risk of SDM.
[37]	To evaluate radiomics models based on T2WI and DWI MRI for predicting pathological complete response (pCR) after nCRT in LARC and compare their performance with visual assessment by radiologists.	Retrospective, single center	898	MRI-based radiomics model showed better classification performance than experienced radiologists for diagnosing pCR in patients with LARC after nCRT.
[38]	To interrogate the mesorectal fat using MRI radiomics feature analysis in order to predict clinical outcomes in patients with LARC.	Retrospective, single center	236	Radiomics features of mesorectal fat can predict pCR and local and distant recurrence, as well as post-treatment T and N categories.
[39]	To develop and validate an AI radiopathomics integrated model to predict pCR in patients with LARC using pretreatment MRI and haematoxylin and eosin (H&E)-stained biopsy slides.	Retrospective, multi-center	303	RAdioPathomics Integrated preDiction System (RAPIDS) was able to predict pCR to nCRT based on pretreatment radiopathomics images with high accuracy.
[40]	To develop and validate a DL model that could preoperatively predict the microsatellite instability (MSI) status of RC based on MRI.	Retrospective, single center	491	DL based on T2WI HR-MRI showed a good predictive performance for MSI status in RC patients.
[41]	To investigate whether DL-based segmentation is feasible in predicting Kirsten rat sarcoma viral oncogene homolog (*KRAS*)/neuroblastoma ras viral oncogene homolog (*NRAS*)/v-raf murine sarcoma viral oncogene homolog B1 (*BRAF*) mutations of RC using MRI-based radiomics.	Retrospective, single center	202	3D V-Net architecture provided reliable RC segmentation on T2WI and DWI compared with expert-based segmentation, and auto segmentation was subjected to radiomics analysis in the prediction of *KRAS*/*NRAS*/*BRAF* mutation status and may produce a good prediction result.
[42]	To build and validate an MRI-based radiomics model to preoperatively evaluate TB in LARC.	Retrospective, multi-center	224	The novel MRI-based radiomics model combining multiple sequences is an effective and non-invasive approach for evaluating TB grade preoperatively in patients with LARC.
[43]	To perform distant metastases (DM) prediction through DL radiomics.	Retrospective, multi-center	235	MRI-based DL radiomics had the potential in predicting the DM of LARC patients receiving nCRT.

### Staging

#### T staging

Nowadays, HR-MRI is considered the most valuable diagnostic method for evaluating RC local extent [9]. However, differentiating which of the parietal layers are involved in the tumour to establish the T stage is a complicated task that can generate staging errors that consequently reflect on therapy [44, 45]. For these reasons, decision support tools based on MRI radiomics and ML have been developed in order to assist radiologists in solving this task [32, 46, 47].

Ma et al. [33] developed an MRI-based radiomics model derived from high-resolution T2WI to discriminate between patients with T1/T2 and those with T3/T4 RC showing sensitivity and specificity respectively of 76% and 74%. In another study, Yin et al. [32] had similar results in differentiating T1/T2 RC forma T3/T4 RC using a logistic regression algorithm applied to DWI in 115 patients, with a sensitivity of 79% and a specificity of 74%.

Furthermore, Lu et al. [46] evaluated the performance of texture analysis using T2WI sagittal fat-suppression combined with axial T2WI using a logistic regression model that reached a sensitivity of 88% and specificity of 61% for discerning between T1/T2 *vs.* T3/T4 [46]. Similarly, in a previous research study, Lu et al. [48] described two radiomics models based on the minimum (tumour only) and maximum (tumour plus blurred area around the tumour) region of interests (ROIs) and applied them to T2WI for the same endpoint. Their models showed an area under the curve (AUC) of 0.808 for the tumour-only ROI and of 0.903 for the maximum ROI [48].

Many researchers have also begun to apply DL to MRI images in order to achieve more precise automatic T staging [49, 50]. Wu et al. [49] explored using a faster region-based CNN (R-CNN) to develop a DL platform based on horizontal, sagittal, and coronal T2WI MRI capable of predicting RC T staging. Their results showed AUCs of 0.95–1.0 for the T1, T2, T3, and T4 stages in the three different planes, highlighting that this AI model could be an effective method for the T staging purpose [49].

Finally, Wang et al. [50] explored the application of the same DL model to evaluate the involvement of circumferential resection margins in HR-MRI. The AI model was trained with 240 RC patients with positive circumferential resection margins in their retrospective study. When tested on the validation group, the AI platform showed an accuracy, sensitivity, and specificity of respectively 0.932, 0.838, and 0.956, with an AUC of 0.953 [50].

#### N staging

Since the lymphatic spread is one of RC’s main important metastatic routes, it is critical to identify pathological nodes before surgery to guarantee LN dissection in these patients [51]. However, conventional HR-MRI has debated a limited role in evaluating LN status in RC, since it provides only information regarding the size, shape, and margins of LNs, thus being characterized by a relatively low specificity [52]. For this reason, the precise evaluation of LN status has become of critical interest. AI has been proposed as a possible tool to improve MRI-based LN assessment in RC via both radiomics and ML algorithms [53]. Recently Ma et al. [33] developed an ML model, associating an random forest (RF) algorithm with radiomics features obtained from T2WI, that could differentiate N0 from N1/N2 stages with a sensitivity and specificity of 79% and 72%, respectively. In another study, Yin et al. [32] received similar good results but with an logistic regression algorithm and an ML model derived from DWI radiomics features.

In another paper, the authors tried to predict the pathological nodal stage of LARC through an alternative radiomics method that exploits collective features of LNs extracted from T2WI acquired before and after nCRT [34]. In detail, they used a training set of 143 patients and a validation set of 72 patients where their logistic regression model predicted pathological node status after nCRT with a sensitivity of 95% and a specificity of 60% in the validation set [34].

Ding et al. [54] built a DL model based on the faster R-CNN to determine the N stage in patients with RC. The authors retrospectively selected MRI images of 414 RC patients discharged from 6 different medical institutes and then applied Faster R-CNN to identify pathological LNs. The results were compared to the radiologists’ scores, with consistency between radiologists and the DL algorithm of 0.912. They concluded that Faster R-CNN is superior to radiologists in the evaluation of metastatic LNs of RC [54, 55].

#### M staging

Finally, the M stage in RC patients is usually established with other diagnostic tools, particularly total body CT. However, a recently published paper demonstrated that the radiomics features obtained from the segmentation of RC could deliver critical data to predict SLM or metachronous liver metastases [35, 56, 57] and SDM to other organs [36]. In particular, Liang et al. [57] used radiomics features extracted from T2WI and post-contrast T1WI dynamic contrast-enhanced (DCE) together with two types of ML models, a support vector machine (SVM) and logistic regression, to predict metachronous liver metastases in 108 RC patients. The algorithm performed slightly better than SVM, with a sensitivity of 83% and a specificity of 76%, suggesting the potential of radiomics to predict which patient will develop liver secondarisms after therapy [57].

Finally, Shu et al. [56] constructed a radiomics nomogram combining clinical risk factors and radiomics features extracted from T2WI MRI images of the primary RC of 194 patients in order to predict the presence of SLM. They used least absolute shrinkage and selection operator (LASSO) and principal component analysis (PCA) models to select the features and then logistic regression and the decision curve analysis algorythms to build the prediction model. Their nomogram showed a good predictive performance with an AUC of 0.912 in the validation set, highlighting the possibility of predicting SLM through radiomics characteristics of the primary tumour [56].

### Predicting treatment response

Surgery, nCRT, or adjuvant chemotherapy are considered the main therapeutical options for patients affected by LARC. As explained above, HR-MRI represents the most valuable tool for tumour assessment for staging, but it is also fundamental for restaging after therapy [6]. However, it did not show great accuracy in discerning fibrotic scars induced by treatments from minimal residual disease [58].

In this setting, the progress of radiomics and AI in diagnostic imaging has paved the way for the development of new tools to assess the treatment response of RC patients [59–61].

Shin et al. [37] created different radiomics models based on T2WI and DWI to predict pCR in a group of 898 LARC patients who underwent nCRT. They used surgical histopathologic analysis as the reference standard for pCR, which was defined as evaluating only the primary tumour. The authors built three models: T2WI, ADC, and a merged one. Among the three models, the T2WI radiomics one showed higher classification performance with an AUC of 0.82 and sensitivity of 80.0% and was superior to experienced radiologists’ performance in diagnosing pCR [37].

Pang et al. [62] projected a system combining radiomics analysis and DL. The model was based on a post-nCRT T2WI MRI, automatically segmenting an ROI on a “suspicious region”, defined as an area with a distinct possibility of containing a tumour or fibrosis as assessed by radiologists. Their method achieved an AUC of 0.815 on the external validation dataset [62].

Jayaprakasam et al. [38] designed a retrospective study to explore the potential of T2WI MRI radiomics feature analysis of the mesorectal fat to predict the nCRT response in 236 patients with LARC. They created a model that could predict pCR with an AUC of 0.89, a sensitivity of 78%, and a specificity of 85.1% [38].

Interestingly, Feng et al. [39] projected the RAPIDS, an ML model based on three feature sets associated with pCR: radiomics MRI features, pathomics nucleus features, and pathomics microenvironment characteristics. Their objective was to predict pCR in patients with LARC starting from a baseline MRI and whole slide images of H&E-stained biopsy slides. Patients had undergone a pretreatment MRI and nCRT followed by surgery. The accuracy of RAPIDS in predicting pCR in LARC was externally validated in two different cohorts with an accuracy of 0.86 and 0.87, respectively. Finally, RAPIDS showed an AUC of 0.812, a sensitivity of 0.888, and a specificity of 0.74 in predicting pCR in the prospective validation study. These results indicated that RAPIDS could represent a novel tool to assist physicians in the tailored management of patients with LARC [39].

Additionally, Aker et al. [63], Yang et al. [64] also evaluated the applicability of texture analysis to multiple MRI sequences to identify potentially significant imaging biomarkers that can accurately detect patients with pCR, highlighting promising results.

Furthermore, some authors also explored the possibility of developing radiomics nomogram to predict pCR [65, 66].

In particular, Wang et al. [65] created an MRI-based radiomics signature to distinguish good responders and poor responders to nCRT and merged it within a nomogram with MRI T stage, ADC values, and circumferential resection margin. Their nomogram could predict a good response to nCRT with a sensitivity of 71% and a specificity of 88% [65]. Similarly, Liu et al. [66] developed a radiomics nomogram based on the combination of a radiomic signature, pre- and post-treatment MRI (T2WI and DWI) images, and the post-treatment tumour length that managed to reach an accuracy of 94% in predicting pCR.

In general, the results of the different models are promising, even if it is not easy to draw a conclusion from the available evidence. However, an early and accurate prediction of the treatment response could significantly improve the management of patients with LARC and favour the development of tailored treatment.

### Genotyping

Radiogenomics represents a recently developed field of imaging whose objective is to obtain genotypic characteristics of a disease through diagnostic images and could optimistically represent a game changer in a radiology-pathology correlation [67].

MSI and *KRAS*/*NRAS*/*BRAF* mutations represent critical, independent prognostic factors in RC patients, and their presence is routinely searched through a genetic test on samples from biopsy or surgery to determine personalised treatment and prognosis [6, 40, 68].

However, biopsy and surgery are invasive, time-consuming techniques with potential complications and depend on specific equipment [40, 41].

Therefore, radiogenomics could represent a non-invasive, low-cost, and time-sparing field of diagnostic imaging that could identify RC genotypic characteristics.

KRAS/NRAS/BRAF are critical proteins in the epidermal growth factor receptor (EGFR) signaling pathway. They control the proliferation, differentiation, and invasion of tumoral cells [69]. Their mutations are responsible for poor response to biological therapy and are linked with a higher risk of developing DM [70, 71]. For this reason, Zhang et al. [72] evaluated the possibility of identifying a radiomics signature from T2WI MRI capable of predicting KRAS status in patients with LARC. The LASSO regression was used to evaluate the associations between the features and gene status. Of the 253 features obtained from T2 images of 83 patients, one feature named X. LL_scaled_std was selected and presented a radiomics-based C-index value of 0.703, suggesting that radiomics features could differentiate KRAS status in these patients [72].

In another paper, Cui et al. [73] used an SVM classifier with T2WI-based radiomics features to evaluate KRAS status in 213 RC patients, providing internal and external validation with an AUC of 0.682 and 0.714, respectively. This result underlines that their radiomics signature could be helpful in predicting KRAS status and may support genomic analysis to establish KRAS expression [73]. Finally, Oh et al. [74] used T2WI MRI-based texture analysis to select three radiomics features significantly associated with *KRAS* mutational status (*P* < 0.05) in patients with RC. The three features were used to create a model with a decision tree to evaluate the presence of *KRAS* mutation. The model comprised four terminal nodes, two of which were able to identify *KRAS* mutation with a sensitivity, specificity, and accuracy of 84%, 80%, and 81.7%, respectively [74].

MSI is a genetic anomaly subsequent to damaged one or more mismatch repair (MMR) proteins [75].

In a recent paper, Zhang et al. [40] explored the feasibility of using an MRI-based DL model, named 3D MobileNetV2 model, to predict MSI status. The group created three models: one based exclusively on clinical factors, one MRI-based, and another combining clinical and imaging characteristics. The imaging-based and the combined model correctly classified 75.0% and 85.4% of MSI status in the test set, with AUC values of 0.820 and 0.868, respectively. Their T2WI MRI-based model showed a good predictive performance for MSI status in RC patients and may represent a helpful tool to select patients who would benefit from chemotherapy or immunotherapy [40].

Furthermore, Li et al. [76] developed a radiomic model based on MRI images to predict preoperative MSI status in RC patients. They created three main models: a T2-based, an ADC-based, and a combined model, which showed an AUC of 0.895, 0.796, and 0.926 in the testing set, respectively [76].

### Assessing high-risk histopathological features

RC may present numerous histopathological features, such as differentiation degree, EMVI, TB, LVI, and PNI that are linked with poor prognosis and have to be taken into account for stratifying the risk of RC patients [5]. A reliable pre-treatment assessment of these factors would probably favour and accelerate the progress of tailored treatment strategies and consequently, the transition toward precision medicine [5, 77–79].

Tumour grading measures cell anaplasia in the sampled tumour and has an essential role in RC prognosis. Well-differentiated tumours generally show better outcomes in this setting [80]. In a recent paper, Meng et al. [81] evaluated the performance of three ML classifiers to recognise well-differentiated RCs on the basis of radiomics characteristics obtained from T2WI, DWI, and DCE MRI sequences. The LASSO algorithm outperformed the other two ML classifiers, with an AUC of 0.72 in the validation group [81]. Furthermore, He et al. [82] developed a feature selection method and ML-based prediction model using MRI-based radiomics features to discern among different pathological grades for RC. The model showed relatively acceptable performance in tumour grading of RC with AUC values of grades 1, 2, 3, and 4 of 0.717, 0.683, 0.690, and 0.827, respectively, in the validation set [82].

TB is an emerging prognostic biomarker in RC. TBs are defined as single cancer cell or clusters of up to four cancer cells located at the invasive tumour front [83]. Recently a group of researchers constructed a model based on multiple MRI-sequences radiomics to identify TB in LARC before surgery, which presented an accuracy of 81.2% [42].

MRI is also considered one the most valuable tool to establish the presence of EMVI; however, HR-MRI performances in evaluating EMVI are still not that high [84].

For this reason, Zhao et al. [85] built a radiomic nomogram based on multiple MRI sequences (T1WI, T2WI, and proton density) to identify EMVI, with an AUC of 0.899, which resulted in being superior to radiologists.

Another interesting nomogram was created by Yu et al. [86], including a DCE MRI radiomics signature and clinical features. They found that the nomogram performed better than quantitative perfusion parameters such as Ktrans in predicting EMVI, with 88.9% sensitivity and 78.3% specificity in the test dataset [86].

Moreover, Shu et al. [87] projected a system based on T1WI, T2WI, DWI, and DCE MRI sequences to detect EMVI. The algorithm was also merged with clinical features and presented an AUC of 0.835, a sensitivity of 0.714, and a specificity of 0.885 [87].

LVI and PNI rapresent two negative prognostic factors in RC. LVI and PNI occur when cancer invades the layers of the lymphovascular wall or spreads along the nerve sheath [88].

Zhang et al. [89] designed a multi-modality radiomic nomogram based on T2WI and DWI MRI sequences plus enhanced CT images to predict the presence of LVI in RC patients. Their nomogram had significant predictive power in the validation cohort with an AUC of 0.876. Instead, Guo and his group [90] operated similarly to project radiomic nomograms to predict PNI in RC patients, obtaining an AUC of 0.884 in the test dataset.

Finally, Chen et al. [91] realised a different radiomics nomogram based only on oblique T2WI MRI sequences that could predict PNI status with an accuracy of 0.71 and an AUC of 0.85.

### Prognosis

Nowadays, although the progress of surgery and CRT protocols have considerably improved the outcomes of patients affected by RC, local recurrence (LR) and DM still represents two critical negative prognostic factors [92, 93]. HR-MRI has a pivotal role in detecting LR during follow-up scans in this setting. Recently, researchers tried to understand the potential of HR-MRI-based radiomics and AI to predict LR in patients with RC [38]. Jayaprakasam et al. [38] developed a T2WI MRI radiomics-based model to predict LR in 236 patients who underwent nCRT. The eight most significative radiomics features were extracted from axial T2WI of mesorectal fat to develop a model using SVM. They found that it could predict LR with a sensitivity and specificity of 68.3% and 80.7%, respectively [38].

DM represents another critical prognostic factor that influences the outcomes of RC patients. Recently, many authors developed different promising radiomic signatures from segmented tumour regions based on multiple HR-MRI sequences that could be used to predict DM-free survival (DMFS) in patients with LARC [94–96].

Moreover, a group of researchers recently designed a retrospective study to explore the capability of an AI radiomics-based model to predict 3-year DMFS in patients with LARC after receiving nCRT, with a C-index of 0.747 and an AUC of 0.894 [43]. Another group built up a radiomic nomogram constructed on an MRI-based radiomic signature that exhibited high performance in predicting DMFS, with C-indices of 0.848, 0.831, and 0.825 in the test groups, respectively [97]. Furthermore, Liu et al. [36] investigated the possibility of predicting SDM in RC patients via a clinical-radiomics model before surgery. They realised a model based on the preoperative T2WI MRI images of 177 patients that showed an AUC of 0.827, which proved that the model could help stratify patients with a higher risk of SDM before surgery [36].

## Limitations

MRI-based radiomics and AI have shown an exciting potential to increase MRI accuracy in T and N staging, to evaluate the patients’ response to nCRT, to obtain genotypic characteristics of the tumour, and to assess prognosis and high-risk variables. However, there is still a long way to go to complete the transition from research to clinical application. First of all, high-quality images are critical to obtaining the best performance from different systems. Since the rectum is a constantly involuntary moving organ, this creates challenges for MRI that has to be solved to improve radiomics and AI models’ performance. Many authors demonstrated that using multimodal imaging and pre- and post-therapy images may be useful to solve this task [39, 98]. ROI segmentation requires trained and skilled operators, which is time- and cost-consuming. In this setting, developing a reliable, fully automated AI segmentation model requires collaboration between engineers and doctors. This AI model should be able to segment precise areas on different MRI sequences rather than only on a single one to speed up the process and save time.

Moreover, the standardisation of radiomics and AI workflow, including the standardisation of the scanning protocol, image reconstruction and preprocessing, and of its evaluation, as well as the validation of the relative models are critical elements affecting the transition towards clinical applications. Furthermore, the currently available models cannot explain the biological meaning of the extracted features. Finally, the majority of studies available developed their algorithm on a small amount of data from a single center [32–36]. Thus, the time has probably come for appropriately designed prospective multicenter trials to build and externally validate reliable MRI-based systems that could guarantee better guidance for the tailored management of patient affected by RC.

## Conclusions

AI models, including radiomics, ML, and DL, have been widely applied in diagnostic imaging. Nowadays, national and international guidelines suggest HR-MRI as the most suitable imaging technique for staging and restaging RC. Recently, many authors explored the feasibility and the potential of these models based on MR images in LARC, mainly to increase its accuracy in T and N staging but also for the evaluation of the patient’s response to nCRT, to obtain genotypic characteristics of the tumour, to assess prognosis and high-risk variables [40–43]. In the next decades, the development of AI systems based on MRI could represent a game changer to support physicians in the transition towards a tailored diagnosis and treatment of RC patients. However, there is still an urgent need to standardise the radiomics and AI workflow, improve efficiency, and primarily design and carry out solid prospective multicenter studies to validate the results of these systems and favour the transition from academic research to the clinical setting.

## Abbreviations

3D: three-dimensional
ADC: apparent diffusion coefficient
AI: artificial intelligence
AUC: area under the curve
BRAF: v-raf murine sarcoma viral oncogene homolog B1
CRT: chemoradiation
CT: computed tomography
DCE: dynamic contrast-enhanced
DL: deep learning
DM: distant metastases
DMFS: distant metastasis-free survival
DWI: diffusion-weighted imaging
EMVI: extramural vascular invasion
HR-MRI: high-resolution magnetic resonance imaging
KRAS: Kirsten rat sarcoma viral oncogene homolog
LARC: locally advanced rectal cancer
LASSO: least absolute shrinkage and selection operator
LNs: lymph nodes
LR: local recurrence
LVI: lymphatic vascular infiltration
ML: machine learning
MRI: magnetic resonance imaging
MSI: microsatellite instability
nCRT: neoadjuvant chemoradiation
NRAS: neuroblastoma ras viral oncogene homolog
pCR: pathological complete response
PNI: perineural invasion
RAPIDS: RAdioPathomics Integrated preDiction System
RC: rectal cancer
R-CNN: region-based convolutional neural network
ROIs: region of interests
SDM: synchronous distant metastases
SLM: synchronous liver metastases
SVM: support vector machine
T2WI: T2-weighted image
TB: tumour budding

## Declarations

### Copyright

@ The Author(s) 2023.
